# Electrochemical Impedance Spectroscopy—A Simple Method for the Characterization of Polymer Inclusion Membranes Containing Aliquat 336

**DOI:** 10.3390/membranes1020132

**Published:** 2011-06-23

**Authors:** Michelle O'Rourke, Noel Duffy, Roland De Marco, Ian Potter

**Affiliations:** 1 Department of Chemistry, La Trobe University, Bundoora, Victoria, 3086, Australia; E-Mails: michelle.roberts@latrobe.edu.au (M.O.); i.potter@latrobe.edu.au (I.P.); 2 CSIRO, Clayton South, Victoria, 3169, Australia; E-Mail: Noel.Duffy@csiro.au; 3 Department of Chemistry, Curtin University, GPO Box U1987 Perth, Western Australia, 6845, Australia

**Keywords:** dielectric constant, polymer inclusion membranes, Aliquat 336, electrochemical impedance spectroscopy, fixed site jumping

## Abstract

Electrochemical impedance spectroscopy (EIS) has been used to estimate the non-frequency dependent (static) dielectric constants of base polymers such as poly(vinyl chloride) (PVC), cellulose triacetate (CTA) and polystyrene (PS). Polymer inclusion membranes (PIMs) containing different amounts of PVC or CTA, along with the room temperature ionic liquid Aliquat 336 and plasticizers such as trisbutoxyethyl phosphate (TBEP), dioctyl sebecate (DOS) and 2-nitrophenyloctyl ether (NPOE) have been investigated. In this study, the complex and abstract method of EIS has been applied in a simple and easy to use way, so as to make the method accessible to membrane scientists and engineers who may not possess the detailed knowledge of electrochemistry and interfacial science needed for a rigorous interpretation of EIS results. The EIS data reported herein are internally consistent with a percolation threshold in the dielectric constant at high concentrations of Aliquat 336, which illustrates the suitability of the EIS technique since membrane percolation with ion exchangers is a well-known phenomenon.

## 1. Introduction

Membranes have been used as a viable alternative to traditional solvent extraction and analytical separation with significant advantages including reduced consumption of reagents and diluents [1]. Accordingly, membranes have gained wider acceptance commercially in membrane filtration, electrodialysis and ion-selective electrodes (ISEs) [2].

Polymer inclusion membranes (PIMs) are used in species transportation, passive sampling and extraction using typically a base polymer, plasticizer, and carrier. The polymer acts as an interconnected support network, while the carrier facilitates species transportation. Plasticizers have a dual role in membranes, since they are used to increase membrane flexibility, species transportation and flux rates (diffusion) and act as a solvent for the carrier within the membrane phase [3]. The desirable properties of a plasticizer are: (1) compatibility with the polymer; (2) low volatility and solubility in aqueous solution; (3) low viscosity; (4) low cost; and (5) low toxicity [1,3]. Too low a plasticizer content results in a brittle and rigid membrane [4]. This embrittlement effect was described by Nghiem *et al.* [4] where they referred to the membrane dielectric constant as the primary factor responsible for membrane anti-plasticization. An ability to control the dielectric constant of the plasticizer and the concomitant membrane [5,6,7] has been pivotal in the successful application of PIMs, and this behavior has been explained theoretically [3].

The PIMs selected for this study are potential extraction, transportation and passive sampling devices for 2,4-dichlorophenoxyacetic acid, or 2,4-D. The carrier used to facilitate the transportation of 2,4-D into the membrane is Aliquat 336, which is a well-known ionic liquid. Aliquat 336 is a mixture of quaternary ammonium chlorides, but predominantly comprises tri-octylmethylammonium chloride.

A number of different techniques including electrochemical impedance spectroscopy (EIS) [8], X-ray photoelectron spectroscopy (XPS) [9,10], and scanning electron microscopy—energy dispersive X-ray (SEM-EDX) [11,12] have been used to characterise PIMs and supported liquid membranes (SLMs) containing ionic liquids. These studies investigated changes in the surface chemistry, such as loss of ionic liquid and micro-emulsion formation, associated with ageing of the membranes.

Studies on the characterization of bulk structural and physicochemical properties resulting from changes in the composition and use of PIMs containing ionic liquids are limited. These properties are influenced by the combination of polymer, plasticiser and carrier that determines the membrane composition and by changes in the membrane brought about by operational and solution conditions. EIS has been used to determine dielectric constants of 4.8 and 13–14 for DOS and NPOE PIMs, respectively, containing 33 wt % PVC and 0.5–5 wt % tridodecylmethylammonium chloride used in potentiometric measurements [13,14], but not for extraction, transportation and passive sampling PIMs containing much higher concentrations of ionic liquid (up to 60 wt %), which is the scope of the present study.

Electrochemical impedance spectroscopy (EIS) may be used in the characterization of membrane capacitance, resistance, dielectric constant, and surface homogeneity. Moreover, EIS has been used previously to determine the membrane bulk resistance [15], as well as the membrane bulk capacitance (Equation 1) [8,16]. The dielectric constant or relative electrical permittivity (Equation 2) is subsequently determined from the measured bulk capacitance and membrane thickness.

(1) $$C=\frac{1}{2\pi fR_{\text{memb}}}$$

(2) $$C=\frac{{ɛ}_{0}{ɛ}_{r}A}{d}$$

where: *C* is capacitance (Farads), *f* is the frequency (at the maximum of the Nyquist plot in Hertz), *R*_memb_ is the resistance of the membrane (Ohms), *ε*_0_ is the relative electrical permittivity of free space (8.854 × 10^−12^ F·m^−1^), *ε*_r_ is the relative electrical permittivity of the membrane, *A* is the area of overlap of the two plates (m^2^ or the exposed membrane area) and d is the distance between the two plates of the capacitor (membrane thickness, m).

Relative electrical permittivity (*ε*_r_) is also known as the static dielectric constant or relative dielectric constant or specific inductive capacity [17]. It is the ratio of stored electrical energy when a potential is applied relative to that of a vacuum. The ability of a material to align its permanent and induced dipoles when an alternating electric field is applied is expressed via the relative static permittivity (*ε*_s_) (Equation 3) [18].

(3) $${ɛ}_{\text{r}}=\frac{{ɛ}_{s}}{{ɛ}_{0}}$$

Static permittivity has contributions from electron and atom polarization, inter-ion polarization, the orientation of permanent dipoles and the Maxwell-Wagner effect [18,19]. The relative static permittivity can be determined experimentally by evaluating the ratio of the capacitance of the dielectric material to that of a vacuum between the plates of a capacitor. The dielectric constant is an important factor when designing a capacitor for electrical circuits, whereas in the context of chemistry, the dielectric constant is a useful measure of solvent and polymer polarity and the concomitant charge carrying ability of a material.

Due to the highly specialized and complex nature of the EIS technique, the authors have developed a simple EIS methodology based on a rudimentary analysis of the bulk membrane resistance and capacitance at high frequency without any complex equivalent circuit fitting, Kramers-Krönig transforms and complex EIS theoretical analysis methods using Equations 1 to 5 for estimation of dielectric constants in membrane materials, so as to make the technique accessible to membrane scientists and engineers. Since the ion-exchangers of these materials can experience ion clustering in the membrane phase, a study of the dielectric constants as a function of composition was deemed appropriate, so as to illustrate that the EIS method may be used in the deduction of information about the structural and physical properties of membrane materials based on the well-known phenomenon of ion-exchanger percolation. Furthermore, we have examined in parallel previously studied membrane materials, as well as ISE membranes to compare and contrast the results, both as a means of experimental validation and to shed new information on molecular aggregation of ion-exchangers in PIM materials.

## 2. Experimental Section

### 2.1. Reagents

Analytical grade reagents of aliquat 336 (Sigma-Aldrich, Castle Hill, New South Wales, Australia), 2-nitrophenyl octyl ether (NPOE) (Sigma-Aldrich, Castle Hill, New South Wales, Australia), dioctyl sebacate (DOS) (Sigma-Aldrich, Castle Hill, Australia), tris(2-butoxyethyl) phosphate (TBEP) (Sigma-Aldrich, Castle Hill, Australia), cellulose triacetate (Sigma-Aldrich, Castle Hill, Australia) (M_n_ 72,000–74,000, 43.6% (w/w) Acetyl), poly(vinyl chloride) (Fluka, selectophore sourced from Sigma-Aldrich, Castle Hill, Australia) (M_n_ 90,000), Polystyrene (Sigma-Aldrich, Castle Hill, Australia), (M_n_ 401,340), dichloromethane (Fluka, HPLC grade sourced from Sigma-Aldrich, Castle Hill, Australia), tetrahydrofuran (THF) (AJAX Chemicals, Cheltenham, Australia), ethyl acetate (AJAX, Chemicals, Cheltenham, Australia), hydrochloric acid (BDH, Kilsyth, Australia) and potassium chloride (Mallinckrodt Baker, Mulgrave, Australia) were used as received, unless otherwise stated.

### 2.2. Methods

Impedance spectra data were acquired using an SI 1287 Solartron Electrochemical Interface (FRA) combined with an electrochemical interface SI 1260 (Solartron Impedance Analyzer) applying an excitation perturbation amplitude of 10 mV root mean square (rms). Zview™ and Zplot™ software version 3.0 were used to record and analyze the impedance data. Measurements were taken over the 0.1–1 MHz frequency range in saturated KCl (Analar) using a four-electrode configuration. Data integration was set at 7 cycles and 13 steps per decade. The cell used for impedance measurements consisted of a symmetrical cell of plexiglass with an exposed membrane diameter of 1 cm and two disk Ag/AgCl electrodes (ø 1.63 cm), and has been described elsewhere in the literature [16,20]. Internal plexiglass mounted Ag/AgCl disks were used as the reference electrodes. Two platinum wires on either side of the membrane (inserted through the solution filling ports of the cell compartments that were central to each compartment) were connected as auxiliary (counter) and working electrodes. The reference electrodes were stripped, polished and recoated before the analysis of each membrane. Discs of 1.77 cm diameter were punched from the parent membrane and placed in the cell. For each membrane composition, three identical disks (triplicates) were examined from the parent membrane and the impedance data were recorded at room temperature (21 °C). Membrane thicknesses were measured 10 times and averaged using a vertically mounted cut membrane edge cross-section by a Nikon Labophot-2 microscope and a Moticam 2300 3.0 MB Camera using Motic Images Plus V2.0 software.

Since the impedance of the PIMs was low, it was necessary to ensure that the solution resistance and charge transfer resistances of the internal reference electrodes were minimized, and made an insignificant contribution to the measured impedance. In this context, the authors used saturated KCl, and this ensured negligible background levels of the solution resistance and charge transfer resistances for the internal Ag/AgCl reference electrodes.

### 2.3. Preparation of Polymer Membranes

A PVC membrane was prepared using 250 mg of PVC high molecular weight selectophore polymer dissolved in 8 mL of THF (distilled under Argon using sodium/potassium amalgam with a benzophenone indicator). Other polymer membranes were prepared as 250 mg of CTA polymer in 8 mL of dichloromethane and 100 mg of polystyrene in 8 mL of distilled ethyl acetate. The homogeneous solutions were poured into a glass ring (4.8 cm diameter) on a glass plate from which the solvent was allowed to slowly evaporate. All other plasticized membrane compositions were prepared to a total mass of 250 mg. PVC membranes contained 40 wt % polymer and various amounts of NPOE plasticizer and Aliquat 336, whereas CTA membranes contained 20 wt % polymer and 0–75 wt % plasticizer (DOS, NPOE or TBEP) and 20–80 wt % Aliquat 336.

## 3. Results and Discussion

### 3.1. Dielectric Constant of Unplasticized Polymer Membranes

Table 1 presents the results for the measured dielectric constants of the non-plasticized polymer membranes and compares them to published literature values [21,22,23,24,25,26]. It is presumed that differences between the values reported herein and literature values are a reflection of the method used to determine the dielectric constant. However, in the authors' opinion, due to the present use of a diffusion cell in the measurement of dielectric constants, the experimental values presented herein are a realistic representation of the experimental conditions in which membranes are utilized in extraction, transportation or passive sampling devices.

**Table 1:** Dielectric constant measurements of polymer membranes including standard deviations obtained from 3 independent EIS measurements.

**Polymer**	**Literature dielectric constant**	**Measured dielectric constant**
PVC	3.3 [22]	13.2 ± 1
CTA	3.6 [26]	21.4 ± 1
Polystyrene	2.45 [23]	9.2 ± 0.8

Furthermore, the literature dielectric constants for PVC were determined using a Schering bridge to measure the electrical capacitance. Although the method used to determine the dielectric constants in the present study also utilized a capacitance measurement, the literature dielectric constants for PVC were determined by extrapolation to a zero concentration of plasticizer solvent for measurements using membranes containing 8–30 wt % of the plasticizer (including, but not limited to, dibenzyl phthalate, dibenzyl maleate, dibenzyl ketone and dibenzyl sebacate). Also, the PVC/plasticizer system was heated to 120 °C in a mould prior to use, and the dielectric constant was determined at specific frequencies [25], as compared to the present method which is frequency non-specific and does not invoke any assumptions about plasticizer effects into the determination of dielectric constant since unplasticized membranes have been studied.

It is important to note that, the average molar mass of PVC used in the literature study was 98,000 g mol^−1^ [25], as compared to 90,000 g mol^−1^ for the PVC used in this work. Accordingly, the 9% difference in average molecular molar mass cannot explain the vast differences between the experimental and literature values for PVC. Most likely, the differences are due to molecular or structural interactions of the polymer, where the measured dielectric constant is dominated by the measurement solvent. The reported dielectric constant for PVC of 3.3–3.5 [27], as measured in dibenzyl sebacate (a plasticizer), which itself has a dielectric constant of 4.6, so the two values are quite close and suggest permeation of the solvent into the membrane.

Similarly, in the literature study of polystyrene, the dielectric constant was determined from a plot of the dielectric constant as a function of polymer concentration with respect to the changing concentrations of polystyrene in a benzene solvent [23]. Polystyrene has a dielectric constant of 2.45 [27] when measured in a benzene solvent, which has a dielectric constant of 2.3.

In both instances, the measured response for the dielectric constant of the polymer is similar to that of the measurement solvent, and this hints at a significant systematic error. This indicates that the dielectric constant of the permeating measurement solvent obscures the dielectric constant for the polymer. The solvent can have a significant effect on the dielectric constant of the polymer due to the energy of interaction between the polymer network and solvent molecules. When the mean energy of interaction between the polymer-solvent exceeds that of the polymer-polymer and solvent-solvent pairs, the polymer will expand further to reduce the contact between adjacent polymer chains [28]. Similarly, Böttcher [17] reports that the *cis*, *trans* and *gauche* position of polymers is dependent upon the organic solvent. Hence, the dominating effect when performing dielectric constant measurements will be the solvent-solvent environment in which the membrane is measured due to the rearrangement of polymer chains in solvents (or in the case of the EIS technique used here, the surrounding solution).

Accordingly, the determination of the dielectric constant of the membranes in aqueous solution, especially the ones plasticized by a hydrophobic solvent, provides a better indication of how the interaction between polymer and the aqueous solution affects the charge carrying ability of the membrane, and this is a major advantage of the EIS method reported herein.

A mass balance of the components in a PVC membrane indicated that there was a negligible amount (<5 wt %) of residual solvent from the membrane casting method trapped within the polymer network. The small amount of residual THF (dielectric constant of 7.58) will have a minor impact on the overall measured dielectric constant of the PVC membranes. The difference between the literature value and the experimental value for the dielectric constant as shown in Table 1 could be due to the influence of the small amount of water dissolved in the THF within the polymer network. However, it is most likely that permeation of water into the polymer chains during EIS measurements is the cause of this discrepancy, as has been reported elsewhere [29]. Water has a dielectric constant of 78.4 at 25 °C, and the dielectric constant of an aqueous solution increases as the ionic strength of the solution increases. Accordingly, since the EIS measurements in this study were performed in saturated KCl solution, the permeation of solvent and salt is expected to elevate the dielectric constants for PVC and polystyrene membranes, as compared to previously reported results [29,30].

**Figure 1:**
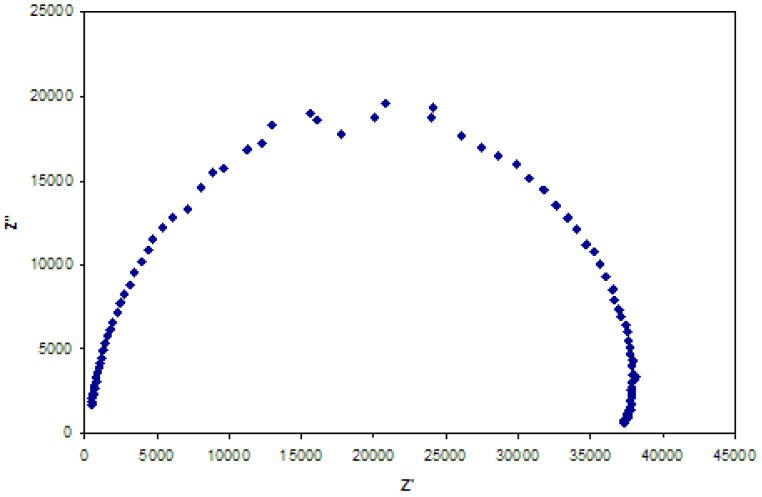
Representative electrochemical impedance spectrum of a poly(vinyl chloride) (PVC) membrane.

**Figure 2:**
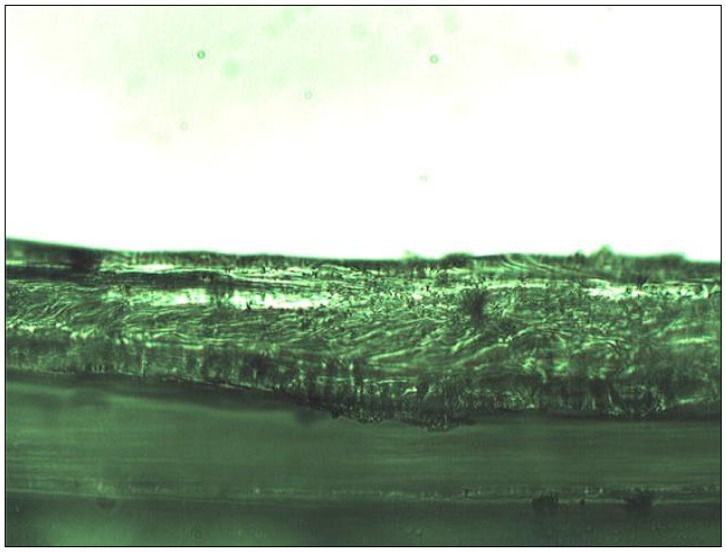
A cut cross-section of a PVC membrane, showing the variation in the membrane thickness. The undulating lower surface is caused by exposure to air and the flat upper surface PVC is in contact to the glass plate when curing.

The frequency dispersion in the measured impedance spectra, as illustrated in Figure 1, shows a compressed semicircle. Frequency dispersion for similar measurements has been reported as being due to a roughened electrode surface [26,30,31,32]; however, the present frequency dispersion is probably due to chemical heterogeneity in the membrane such as polymer and plasticizer domains, but may also be ascribable to variations in the thickness of the solvent cast membranes. Note that it has been reported that, during membrane casting while the solvent evaporates slowly from the homogeneous solution, the membrane surface exposed to air is always rougher than the surface contacted to the glass [33], it was found in this work that the thickness of the PVC membrane varied (10%) along a cut cross section (see Figure 2). These variations in the membrane thickness manifested themselves in the EIS spectra as the observed compression of the semicircle in the Nyquist plots. Consequently, a determination of the capacitance and dielectric constant necessitated the introduction of a constant phase element (CPE) parameter in the equivalent circuit fitting of EIS data. Zview™ software was used to calculate the estimated capacitance via Equations 4 and 5 [34].

(4) $$Z(\text{CPE})=Z_{\omega }{\left(j\omega \prime \right)}^{-\alpha }$$

(5) $$C=\frac{{\left({\omega \prime \prime }_{m}\right)}^{\alpha -1}}{Z_{0}}$$

where, Z_0_ is the magnitude of the impedance, *ω* is the angular frequency, *j* is the imaginary axis, *ω*′ is the phase angle between the applied voltage and current intensity, ω″_m_ is the radial maximum frequency of the imaginary axis (Hz) and *α* = 0 for an ideal resistor and *α* = 1 for an ideal capacitor.

Armstrong *et al.* [35] used a method similar to the one reported in this paper to determine the dielectric constant of PVC. They used a homogeneous PVC membrane and a four-electrode configuration in 0.1 M KCl solution. Their result for the dielectric constant of PVC (4.1) is similar to that reported for PVC of 3.3–3.5 [27]. The variation in the dielectric constant values for PVC between Armstrong *et al.* [35] and those of this study could be due to the value of variable A used in Equation 2, as well as the permeation of saturated KCl with the water permeating into the membrane. In the present study, the value of A is interpreted as the actual surface area of the membrane exposed to the aqueous solution, as the membrane is effectively the “capacitor plate”. By contrast, Armstrong *et al.* [35] defined the value A as the geometrical area of the electrode, without specifying the particular electrode, so this may be a source of experimental uncertainty.

### 3.2. Dielectric Constant of PIMs

EIS studies of ISE membranes have been previously measured to garner information about the capacitance and charge transfer resistance [8,15,16,36,37] rather than the dielectric constant of the membranes. Additionally, previous studies on the dielectric constant of polymer membranes have not included ionic liquids. However, in the previous ISE studies, some of which are discussed below, there are similarities in the data reported in this paper, especially regarding the bulk membrane resistance and the observed differences in membrane resistances in the presence of different plasticizers.

A typical ISE membrane consists of 30–33 wt % polymer (PVC), 60–66 wt % plasticizer, and 1–10 wt % ion-exchanger or neutral carrier. By contrast, PIMs usually contain significantly more ion exchanger up to 80 wt % [19]. The result of such a high concentration of ion exchanger is a considerably higher number of ionic sites. This gives the membrane a significantly diminished resistance and capacitance, as evidenced by a low impedance response. In some cases, inductive behavior is observed, which suggests a resistance to phase change to the frequency scanning of the membrane. As a result, obtaining a phase shift to determine the capacitance and resistance of the membrane is extremely difficult, particularly from inductive elements that appear in the high frequency range (>100 kHz). These may be due to instrument, electrode or connecting wire artifacts [38,39,40]. With the exception of the NPOE membrane series, the resistance of the measured membranes containing more than 30 wt % Aliquat, were too small to determine the geometric capacitance and dielectric constant, and these determinations were therefore avoided.

Plasticized membranes used for the dielectric constant measurements were mostly CTA membranes; however, a few PVC membranes were tested for comparison with the results from Muslinkina *et al.* [16]. In the membrane EIS studies of PVC calcium ISEs by Muslinkina *et al.* [16], it was shown that the bulk membrane resistances for PVC membranes of similar composition but containing different plasticizers, have much lower resistances with NPOE than with DOS. In this study, the resistance of a TBEP membrane was the same as a NPOE membrane (290 Ω) for a composition 20 wt % CTA, 30 wt % Aliquat and 50 wt % plasticizer. When NPOE and TBEP membranes were compared to a similar membrane composition containing DOS as the plasticizer, the measured resistance was higher at 731 Ω, comparable to the results reported by Muslinkina *et al.* [16].

Membranes prepared without plasticizers were also investigated. The results presented in Table 2 indicate that, above 20 wt % Aliquat 336, the dielectric constant increases significantly, and a dramatic increase is observed at 50 wt % Aliquat.

**Table 2:** Dielectric constant of CTA membranes including standard deviations obtained from 3 independent EIS measurements. Percentages listed are wt %.

**NPOE membrane composition**	**Dielectric Constant**
20% CTA, 20% Aliquat, 60% NPOE	54 ± 6
20% CTA, 30% Aliquat, 50% NPOE	80 ± 2
20% CTA, 35% Aliquat, 45% NPOE	2,500 ± 300
20% CTA, 40% Aliquat, 40% NPOE	5,200 ± 350
20% CTA, 50% Aliquat, 30% NPOE	5,200 ± 130
**DOS membrane composition**	
20% CTA, 10% Aliquat, 70% DOS	9.9 ± 0.6
20% CTA, 20% Aliquat, 60% DOS	8.8 ± 1
20% CTA, 25% Aliquat, 55% DOS	29 ± 3
20% CTA, 30% Aliquat, 50% DOS	42 ± 1
**TBEP membrane composition**	
20% CTA, 5% Aliquat, 75% TBEP	320 ± 30
20% CTA, 10% Aliquat, 70% TBEP	6500 ± 500
20% CTA, 20% Aliquat, 60% TBEP	26,000 ± 2,000

In previous studies, membranes suitable for the transportation and extraction of analytes containing a plasticizer and Aliquat 336 were also investigated. A study by Sodaye *et al.* [3] reported a dielectric constant of 3.5 for a membrane of composition 36 wt % CTA, 55 wt % NPOE and 9 wt % Aliquat 336. The dielectric constant was calculated using the Born-Haber cycle that is used to determine reaction energies of metal ionic compounds. However, this method does not take into account the hydration and swelling of the membrane, nor the solvated ions that would increase the dielectric constant of the PIM.

Membranes of PVC, Aliquat and NPOE were prepared in order to compare the values of the dielectric constant with those determined by Armstrong *et al.* [37] (Table 3). These membranes contained double the polymer concentration compared to the CTA membranes, as this is the minimum amount required to make a mechanically stable membrane. The results demonstrated that the dielectric constants for PVC membranes are similar to CTA membranes containing the same concentration of Aliquat. This result suggests that the primary influence on the dielectric constant for these membranes is the ionic liquid, Aliquat. In the study of Armstrong *et al.* [37], the dielectric constant for a membrane containing 33 wt % PVC plasticized with 66 wt % DOS, was 4.8 compared to a dielectric constant of approximately 9 for a membrane plasticized with NPOE [37]. Similarly, the results reported herein also indicate that a membrane plasticized with 50 wt % NPOE gives a higher dielectric constant than a similar membrane containing DOS, the values being 80 and 42, respectively.

**Table 3:** Dielectric constants of PVC membranes with Aliquat 336 and NPOE as plasticizer including standard deviations obtained from 3 independent EIS measurements. Percentages listed are wt %.

**PVC membrane composition**	**Dielectric constant**
40% PVC, 20% Aliquat, 40% NPOE	24 ± 3
40% PVC, 30% Aliquat, 30% NPOE	52 ± 15
40% PVC, 40% Aliquat, 20% NPOE	2200 ± 200

In the NPOE membranes, the dielectric constant shows little variation until 30 wt % Aliquat 336 is reached when the dielectric constant rises abruptly. A likely explanation, as proposed by Riggs and Smith [41], involves a “percolation threshold” and fixed site jumping mechanism. The carrier molecules are thought to act like “stepping stones” inside the membrane. Prior to the percolation threshold, the fixed sites are not close enough for the target species to jump from one site to another. One interpretation is that the Aliquat aligns itself to stabilize its dipole at opposite ends when the perturbation is applied, which is similar in organization to that described for a fixed site jumping mechanism [41,42,43]. An alternate explanation is that at high levels of Aliquat concentration, the ionic liquid solvates itself by forming aggregates [44].

It is important to note that the polystyrene/Aliquat membranes did not yield stable and reproducible EIS behavior, so the dielectric constants in these membranes could not be measured reliably, and are therefore not reported in the present paper. Nevertheless, unplasticized polystyrene had been used successfully, as reported in Table 1, as a test of the validity of the EIS technique by a comparison of measured and literature data.

In the absence of a plasticizer, a percolation threshold was observed at 20 wt % Aliquat 336. For membranes containing NPOE as an additional plasticizer, a maximum dielectric constant corresponding to a percolation threshold is reached at 40 wt % Aliquat 336.

The dielectric constant for membranes containing different concentrations of Aliquat and DOS are shown in Table 2. The results indicate that there is a percolation threshold at 20 wt % Aliquat, and an abrupt rise in dielectric constant after that, corresponding to an increase in the level of Aliquat 336. Unfortunately, membranes that contained more than 30 wt % Aliquat showed inductive behaviour or very low resistances and capacitances, which inferred an excessively high dielectric constant. Consequently, their dielectric constants could not be accurately measured. Compared to the membranes containing TBEP and NPOE, membranes of similar composition with DOS gave lower values for the dielectric constant.

The results for membranes containing TBEP (Table 2) show a much higher dielectric constant for the same amount of Aliquat when compared to membranes with other plasticizers. Dielectric constants for TBEP and NPOE have been reported and while TBEP has a low dielectric constant of 3.9 [35], 4.2 [45] and 8.7 [30] compared to NPOE of 23.1 [30] and 24 [6], both have a similar viscosity; 12.2 and 13.8 cP respectively. It is likely that Aliquat arranges itself into a fixed site dipole arrangement at a much lower Aliquat concentration for TBEP compared to other plasticizers. Unlike DOS and NPOE, the membranes containing TBEP do not show a clear percolation threshold. Membranes containing TBEP at an Aliquat content in excess of 30 wt % did not have a measureable resistance or capacitance, but exhibited inductive behavior.

In comparing the dielectric constant for similar membranes of composition 20 wt % CTA, 20 wt % Aliquat and 60 wt % plasticizer, there was no obvious explanation to account for the differences between the plasticizers in the PIM measured dielectric constants. It appears that the most significant factor in the measured dielectric constant of the PIMs investigated in this study, was the ionic liquid, Aliquat 336, and its physical rearrangement.

It should be pointed out that plasticized membranes containing high amounts of Aliquat (*i.e.*, 50–60 wt %), although stable during initial EIS measurements, became unstable with time, and this behavior was associated with an “oily” appearance to the membrane surface, which may also be attributable to exudation of the ionic liquid from the membrane surface. This phenomenon is interesting and requires either an electron microscopy or atomic force microscopy study to identify the origins of the unusual behavior, but such a study was not undertaken as it was considered to be beyond the scope of this study into the dielectric constants of PIMs.

**Figure 3:**
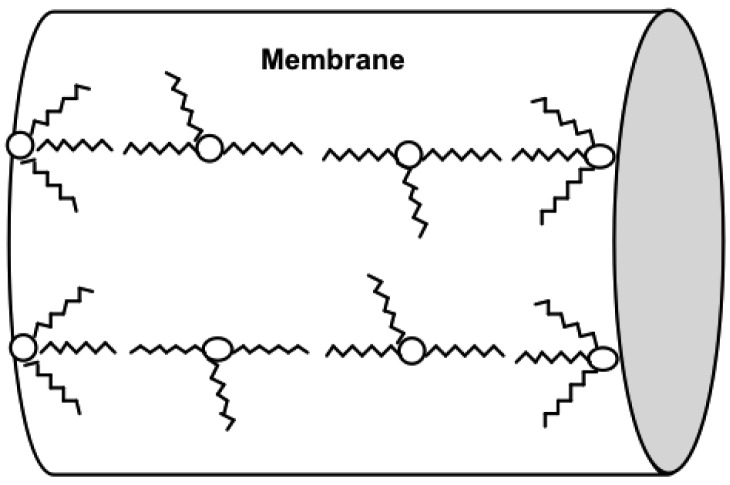
The charge density stabilization of Aliquat 336 in a thin membrane immersed in saturated KCl solution. The Aliquat chloride forms an interface on the membrane surface [46]. Reverse micelles of Aliquat 336 are formed inside the membrane (which also occurs at high concentrations of Aliquat 336).

Aliquat units will be arranged in the membrane so as to minimize the charge density of ions when a perturbation is applied [44], an effect leading to a disproportionately high dielectric constant. This is readily apparent at high concentrations of Aliquat 336. This effect is also observed at a low Aliquat content in thin (<75 μm) membranes, because the membrane is affected strongly by the polar aqueous solution. The Aliquat ion-pairs need to stabilize (Figure 3) within the physical membrane structure when a perturbation is applied. Aliquat 336 is a surface active compound and will orient itself so that ammonium head groups face the water at the membrane-solution interface, while the long alkyl chains will face away from the interface and into the membrane [46].

The mechanism by which the Aliquat stabilizes itself within the membrane is proposed to be a linear arrangement of aggregates or inverted micelles. Amine extractants have been reported by Herriott *et al.* [47] to form aggregates of inverted micelles with a small degree of aggregation (three to seven molecules). Further investigations by Dorbritz *et al.* [44] on the aggregate formation of ionic liquids using mass spectrometry found that ionic liquids would aggregate (like micelles) in solution. They found that the tendency to form aggregates increased as the polarity of the solvent decreased. This effect occurs so as to minimize the charge density of ions. The outer solution has high polarity, while the inner membrane, by comparison, is highly hydrophobic. Accordingly, the work by Dorbritz *et al.* [44] demonstrated that the dielectric constant of the surrounding liquid varies according to the structural arrangement of the ionic liquids.

The aggregate formation of the ionic liquid is the most likely explanation for a structural arrangement that explains the relationship between the Aliquat content and the high dielectric constant. This effect was observed by Riggs *et al.* [41] for the transport of saccharides in PIMs. They calculated that the percolation threshold translated to a distance of about 14 Å between adjacent trioctylmethylammonium chloride (TOMA-Cl) ion-pairs. Smith *et al.* [41] proposed a mobile fixed-site jumping mechanism for the transport of carbohydrates [41,43,48]. A mobile or fixed-site jumping mechanism shows the TOMA-Cl in a linear arrangement that is similar to the proposed linear reverse micelle arrangement (Figure 3).

**Figure 4:**
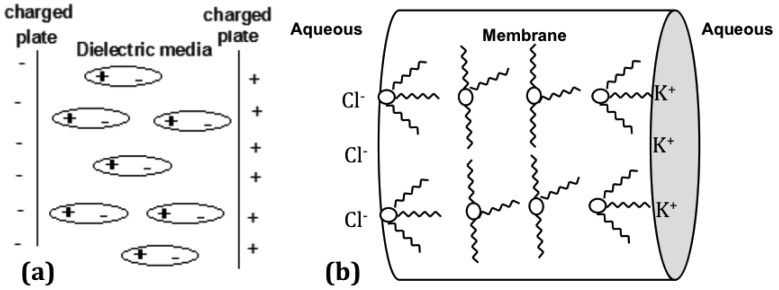
Parallel plate capacitor with an applied voltage, shows the dielectric medium polarized in the opposite direction to the parallel plates **(a)**; and the effect on the membrane when a frequency perturbation is applied to the internal dynamics of the ionic liquid Aliquat 336 **(b)**. The energy applied to the membrane is stored like a capacitor by the rotation of the dipole in the Aliquat molecules to the opposite polarity of the edges of the membrane.

The parallel plate capacitor or metal plate capacitor serves as a good explanation to understand the nature of the linear arrangement of the ion pairs in the membrane and the effect on the dielectric constant. A simple capacitor consists of two metal plates with a conductive material in between. An applied voltage induces one plate to become negatively charged, while the other is positively charged. The dielectric material between the two metal plates creates an electric field in the opposite direction of the charge on the plates. This reduces the overall electrical field between the plates, allowing a higher charge to be stored on each plate [49]. In Figure 3, the Aliquat is depicted in a reverse micelle arrangement, which would be the lowest energy state for the system. However, when a frequency perturbation is applied, the dielectric material will align in the presence of the electric field as shown in Figure 4a [50]. The dielectric material in a capacitor minimizes the electrostatic repulsion forces of the charged plates. More charge can be stored for each plate on either side of the capacitor when the thickness of the dielectric material is increased [49].

The high dielectric constant of the membrane is equivalent to its ability to hold more charge on either side of the membrane. This higher charge is enabled because of the orientation of the Aliquat units from a reverse micelle formation to the typical charge separation seen in a parallel plate capacitor, as shown in Figure 4b.

When Aliquat is present in low concentration in a membrane of standard thickness, the distance between neighboring Aliquat units is greater than the percolation threshold of 14 Å, and the Aliquat units cannot interact with each other to form a reversed micelle arrangement. Consequently, at the membrane-solution interface, the Aliquat is unable to line-up with the applied electric field to prevent the repulsion of electrostatic forces of the charged plates. The Aliquat at low concentrations is therefore trapped within the polymer chains, and is unable to move freely to form a linear arrangement. However, in a thin membrane (<75 μm) with a low Aliquat 336 concentration, or a thick membrane containing a high concentration of Aliquat 336, the neighboring Aliquat units are within a separation of 14 Å, and can form a linear reversed micelle arrangement that gives a high dielectric constant. The reversed micelle arrangement of the Aliquat units requires kinetic energy to align in the opposite direction to the charged plates. This extra energy translates as a greater charge build up on the membrane edges, thereby giving a high dielectric constant.

## 4. Conclusions

The results for the dielectric constant measurements for all of the different membrane compositions showed two similarities. Firstly, there is a non-linear change in the measured dielectric constant as the plasticizer content is increased, or when there is a decrease in Aliquat content. It was shown that, with low dielectric materials like the polymers PVC and CTA, the dielectric constant of a membrane is predominantly influenced by the dielectric constant of the solution in which it is measured. Secondly, two of the plasticizers, NPOE and DOS, exhibited a well-defined plasticization effect, whereby the value for the dielectric constant was fairly constant up to a critical Aliquat concentration known as the percolation threshold, after which there was a dramatic increase by several orders of magnitude. Other studies determined the values of dielectric constants of these ionic liquids by an extrapolation method that is used to determine the dielectric constant of electrolyte solutions [51]. They found that these ionic liquids have a dielectric constant between 10–30 [52]. However, in the present study of PIMs, dielectric constants higher than 5,000 have been measured. These results cannot be attributed to the surrounding solution, or to the dielectric constant of the room temperature ionic liquid (RTIL), Aliquat 336. Rather, the high dielectric constant values indicate the formation of aligned molecular aggregates of Aliquat in the PIM.

The plasticizer can influence the availability of Aliquat in the membrane that results in a high dielectric constant. This occurs when the Aliquat forms a mobile fixed site structure [42] or linear reversed micelle arrangement. In the absence of a plasticizer, 50 wt % Aliquat is needed to form reverse micelles in the membrane, whereas in the presence of the plasticizers NPOE and DOS, the formation of reversed micelles occurs at 35 wt % and 30 wt % Aliquat, respectively. For TBEP, a significant increase in dielectric constant occurs at 10 wt % Aliquat. This suggests that, in the presence of TBEP, a small amount of Aliquat can form a mobile fixed-site. These results cannot be attributed to the dielectric constant of the plasticizer. It has been hypothesized that the correlation between the static dipole moment and the induced dipole moment (measured dielectric constant) is not necessarily the same. In cases of polymer associations, the induced dipole can be greater than the static dipole, which is associated with a physical process or energy change. Whatever the reason for this unusual behavior in the dielectric constants, it is clearly related to the molecular orientation of charge carriers in the membrane phase, and an exact structural study of this phenomenon was beyond the scope of this focused study into the development of an EIS method for the determination of dielectric constants in PIM membrane materials, along with the influence of membrane composition on membrane dielectric constants under realistic conditions of membrane transportation, extraction and passive sampling for which these membranes have been designed.
